# Effect of ambient temperature on overweight: direct and indirect pathways in a multiple mediation model

**DOI:** 10.1590/0102-311XEN090225

**Published:** 2025-12-01

**Authors:** Julia Mariel Wirtz Baker, Laura Rosana Aballay, Alberto Rubén Osella, Victoria Lambert, Sonia Alejandra Pou

**Affiliations:** 1 Instituto de Investigaciones en Ciencias de la Salud, Consejo Nacional de Investigaciones Científicas y Técnicas/Universidade Nacional de Córdoba, Córdoba, Argentina.; 2 Centro de Investigaciones em Nutrición Humana, Universidad Nacional de Córdoba, Córdoba, Argentina.

**Keywords:** Climate, Overweight, Mediation Analysis, Physical Activities, Clima, Sobrepeso, Análisis de Mediación, Actividad Física, Clima, Sobrepeso, Análise de Mediação, Atividade Física

## Abstract

Global warming and obesity are two major global challenges. The intricate relationships between climate, lifestyle factors, and their combined impact on overweight remain to be fully elucidated. We aim to estimate the effect of ambient temperature on overweight and examine the role of physical activity and fruit/vegetable consumption as indirect mediating pathways in Argentina. This cross-sectional study was conducted using data from the 2018 *National Risk Factors Survey*. Average air-temperature at 2m height (from ERA5 reanalysis data) was linked with individual-level data. A multilevel logistic generalized structural equation model was applied to examine the mediating effects of physical activity level and fruit/vegetable consumption on the association between ambient temperature and overweight, adjusted for sex, age, and educational level. The raw difference (95%CI) between the indirect effects was estimated using bootstrapping techniques (sample = 10,000, replicates = 5,000). An inverse association (direct effect) was observed between ambient temperature and overweight (c = -0.019, 95%CI: -0.034; -0.004). A one-unit increase in temperature was associated with higher log odds of fruit/vegetable consumption (a_1_ = 0.020, 95%CI: 0.005; 0.035) and lower log odds of having moderate (a_21_ = -0.015, 95%CI: -0.023; -0.007) and high (a_22_ = -0.059, 95%CI: -0.068; -0.049) physical activity levels, compared to low fruit/vegetable consumption and low physical activity level, respectively. However, the mediating effect of high physical activity level on the temperature-overweight relationship was of greater magnitude. In conclusion, ambient temperature influences fruit/vegetable consumption and physical activity, indirectly affecting nutritional status, with physical activity acting as the key mediator. This underscores the need to prioritize climate change adaptation strategies that promote physical activity.

## Abstract (es)

El calentamiento global y la obesidad son dos grandes retos mundiales. Las complejas relaciones entre el clima, los factores relacionados con el estilo de vida y su impacto combinado sobre el sobrepeso aún no se han aclarado por completo. Nuestro objetivo fue estimar el efecto de la temperatura ambiente sobre el sobrepeso y examinar el papel de la actividad física y el consumo de frutas y verduras como posibles vías mediadoras indirectas en Argentina. Este estudio transversal se llevó a cabo utilizando datos de la *Encuesta Nacional de Factores de Riesgo* de 2018. La temperatura media del aire a 2m de altura (a partir de los datos de reanálisis ERA5) se vinculó con los datos a nivel individual. Se aplicó un modelo logístico multinivel de ecuaciones estructurales generalizado para examinar los efectos mediadores del nivel de actividad física y el consumo de frutas y verduras en la relación entre la temperatura y el sobrepeso, ajustado por sexo, edad y nivel educativo. Se calculó la diferencia bruta (IC95%) entre los efectos indirectos utilizando técnicas de *bootstrapping* (muestra = 10.000, repeticiones = 5.000). Se observó una asociación inversa (efecto directo) entre la temperatura y el sobrepeso (c = -0,019, IC95 % -0,034; -0,004). Un aumento de una unidad en la temperatura se asoció con una mayor chance logarítmica de consumo de frutas y verduras (a_1_ = 0,020, IC95%: 0,005; 0,035) y una menor chance logarítmica de tener una actividad física moderada (a_21_ = -0,015, IC95%: -0,023; -0,007) y alta (a_22_ = -0,059, IC95%: -0,068; -0,049), en comparación con un bajo consumo de frutas y verduras y un bajo nivel de actividad física, respectivamente. Sin embargo, el efecto mediador del alto nivel de actividad física en la relación entre la temperatura y el sobrepeso mostró una magnitud mayor. En conclusión, la temperatura ambiente influye en el consumo de frutas y verduras y en la actividad física, lo que afecta indirectamente al estado nutricional, siendo la actividad física el mediador clave. Esto subraya la necesidad de centrar los esfuerzos en estrategias de adaptación al cambio climático que promuevan la actividad física.

## Abstract (pt)

O aquecimento global e a obesidade são dois grandes desafios globais. As relações intrincadas entre o clima, fatores de estilo de vida e seu impacto combinado no sobrepeso ainda precisam ser elucidadas. Nosso objetivo foi estimar o efeito da temperatura ambiente no sobrepeso e examinar o papel da atividade física e do consumo de frutas/vegetais como vias de mediação indireta na Argentina. Realizou-se estudo transversal utilizando dados da *Pesquisa Nacional de Fatores de Risco* de 2018. A temperatura média do ar a 2m de altura (dos dados de reanálise do ERA5) foi relacionada a dados individuais. Um modelo de equações estruturais generalizadas logístico e multinível foi aplicado para examinar os efeitos de mediação do nível de atividade física e do consumo de frutas/vegetais na relação entre temperatura e sobrepeso, com ajustes por sexo, idade e nível educacional. A diferença bruta (IC95%) entre os efeitos indiretos foi calculada, empregando técnicas de *bootstrapping* (amostra = 10.000, réplicas = 5.000). Associação inversa (efeitodireto) foi observada entre temperatura e sobrepeso (c = -0,019, IC95%: -0,034; -0,004). O aumento de uma unidade na temperatura foi associado a maiores chances logarítmicas de consumo de frutas/vegetais (a_1_ = 0,020, IC95% 0,005; 0,035) e menores chances logarítmicas de ter nível de atividade física moderado (a_21_ = -0,015, IC95% -0,023; -0,007) e alto (a_22_ = -0,059, IC95%: -0,068; -0,049) quando comparado ao baixo consumo de frutas/vegetais e ao baixo nível de atividade física, respectivamente. No entanto, o efeito mediador do alto nível de atividade física na relação temperatura-sobrepeso apresentou magnitude maior. Em conclusão, a temperatura ambiente influencia o consumo de frutas/vegetais e a pratica de atividade física, afetando indiretamente o estado nutricional, com a atividade física atuando como o principal mediador. Isso reforça a necessidade de concentrar esforços em estratégias de adaptação às mudanças climáticas que promovam a atividade física.

## Background

Overweight is a significant risk factor for mortality and morbidity, responsible for about 3 million deaths each year worldwide ^1^. The rising prevalence of overweight can be attributed to an increase in individual energy intake, coupled with a decline in energy expenditure resulting from an unhealthy diet and sedentary lifestyle. However, it is important to recognize that genetic, biological, and environmental factors may also contribute to this phenomenon ^2^. Few studies have investigated how climate variables can impact body mass index (BMI), weight gain, and obesity ^3^.

The recognition of obesity and overweight as two of the greatest public health challenges of this century is accompanied by growing concern over the health impacts of climate change on populations. A global syndemic of obesity, undernutrition, and climate change has been identified ^4^, in which climate variations − particularly rising ambient temperatures − increase the risk of food insecurity by impacting food quality, availability, accessibility, and affordability ^5^. Climate variations also pose increasing risks during physical activity ^6^. However, the mechanisms underlying the complex relationships between ambient temperatures, diet, and physical activity, and their impact on the nutritional status of populations, have not yet been fully explained.

Recent studies have examined the relationship between temperature and food intake ^7^ ^,^ ^8^. Some findings suggest that weather conditions can influence consumer decisions, indicating that high temperatures impair body thermoregulation, reducing food demand ^7^. Additionally, from a climate change perspective, it has been highlighted that long-term climate variations directly impact food systems and, consequently, global food production ^9^. These changes could directly influence human nutrition and health ^10^. 

Environmental conditions can either provide or limit opportunities for physical activity. Studies have shown that extreme weather conditions, particularly heat stress, can reduce mobility and willingness to engage in physical activity ^10^ ^,^ ^11^. The frequency and duration of extreme temperature events have been shown to directly and negatively impact physical activity patterns over the medium and long term ^12^. However, a knowledge gap remains regarding the specific weather conditions that lead to less favorable physical activity patterns, and how these conditions interact with dietary habits. 

Recently, epidemiological research has gone beyond the “black box” exposure-outcome paradigm ^13^ ^,^ ^14^, and one effective strategy involves assessing mediation ^15^. Mediation analysis enables researchers to probe underlying mechanisms within a causal relationship, providing evidence to test path-specific hypotheses. Moreover, it offers valuable insights for designing and refining targeted interventions ^16^.

The application of mediation analysis would contribute to advancing scientific knowledge on the intersection between climate and health, which is particularly important for Latin America, where significant methodological gaps regarding climate analysis and health data have been recently identified ^17^. In this regard, it is noteworthy that Argentina has a valuable data source to explore the nutrition-climate relationship further: the *National Risk Factors Survey* (*Encuesta Nacional de Factores de Riesgo* − ENFR).

The ENFR is a nationwide survey conducted regularly to examine common and known risk factors for chronic diseases, including physical activity and fruit/vegetable consumption. While the relationships between behavioral factors and obesity have already been studied using this data source ^18^, the effects of temperature on them have yet to be explored. 

In this context, we hypothesized that the effect of temperature on overweight could be partially mediated by physical activity levels and fruit/vegetable consumption. By using multiple mediator models, it is possible to simultaneously test and compare different indirect effects. Distinguishing the role of these mediating pathways could be particularly useful for helping decision-makers design public health policies ^19^. 

This study aims to estimate the effect of ambient temperature on overweight and examine the role of physical activity and fruit/vegetable consumption pathways as mediating mechanisms in the Argentine adult population of 2018.

## Methods

### Study design and data

A nationwide cross-sectional study (n = 16,410) was conducted in Argentina using secondary data from the ENFR, conducted in the last quarter of 2018 by the Argentine Ministry of Health and the National Institute of Statistics and Census. The objective of the ENFR is to provide information on risk factors for noncommunicable diseases (NCDs) in Argentina. The ENFR used a probabilistic, multistage sampling design, the details of which have been described elsewhere ^18^. The initial sample comprised 29,224 individuals who completed the first part of the questionnaire, covering all jurisdictions nationwide. For the second stage, which included anthropometric measurements, a probabilistic subsample was drawn from 75% of the selected households. Out of a total of 23,556 households who completed the questionnaire, 16,577 individual interviews were conducted (non-response rate of 21%). This sample is representative of the population aged 18 years and older living in Argentine urban areas with at least 5,000 inhabitants ^20^. For our analysis, we used a subset of 16,410 individuals aged 18 or older with complete and consistent anthropometric data. We excluded 167 cases that did not meet these inclusion criteria due to missing or inconsistent information.

The ENFR uses a questionnaire integrated into a mobile data collection device, administered by trained interviewers and health professionals responsible for physical measurements. This questionnaire includes individual anthropometric data (weight and height), sociodemographic characteristics at the household and individual level, and lifestyle-related data such as physical activity level (measured in total daily metabolic equivanlent − METs) and fruit/vegetable consumption. Height and weight were measured by a trained health personnel using portable equipment, following the World Health Organization (WHO) STEPS protocol endorsed by Argentine Ministry of Health and Social Development ^18^. In this study, BMI was categorized as overweight (BMI ≥ 25, no/yes) based on the WHO criterion ^21^. Physical activity was categorized into three levels according to the *International Physical Activity Questionnaire* (IPAQ) criterion (low, moderate, and high) ^22^. Fruit and vegetable consumption was assessed via a closed-ended question administered by a trained interviewer, asking the respondent about the number of portions consumed on a typical day. In this study, we used the consumption variable from the ENFR dataset, categorized as adequate (≥ 5 servings/day) or inadequate, in accordance with the dietary guidelines for the Argentine population ^23^. 

In addition, ambient temperature data were extracted using the Google Earth Engine (https://earthengine.google.com/) geospatial platform. Specifically, ERA5 images were filtered by date and location, and the average air temperature at 2m height (mean_2m_air_temperature variable) was obtained from the *ERA5 Monthly Aggregate* temperature data file for 2018 ^24^. The raster layer representing annual means was imported into QGIS software (https://qgis.org/en/site/), where the mean pixel values were calculated for each province (n = 24 provinces/jurisdictions) to obtain the average annual temperature at the provincial level. This environmental information was combined with individual data, considering the geolocation of residences at the provincial level. Training was provided in the extraction and processing of environmental data, and the research team employed a double control system.

### Ethical considerations

All procedures were performed in compliance with relevant laws and institutional guidelines. Since this study was based on already available and anonymized secondary data sources, ethical approval and informed consent were not required.

### Statistical analysis

#### Data description

**Figure 1:**
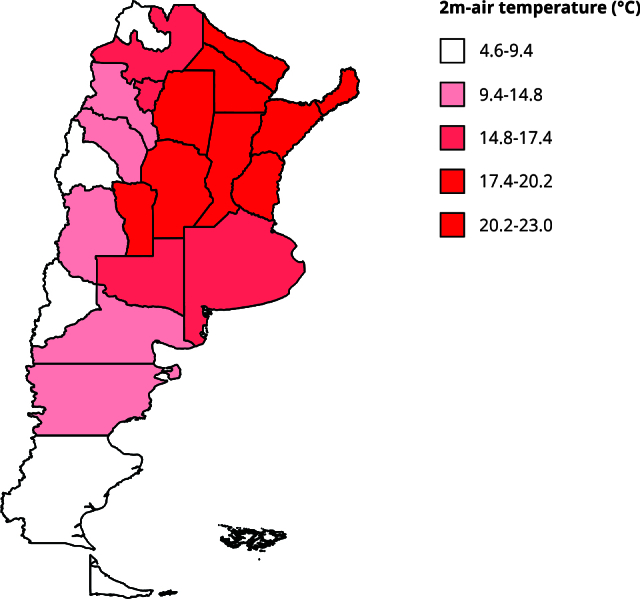
Distribution of the annual mean of 2m-air temperature (°C) by provinces in Argentina, 2018.

Note: the map shows the Argentine provinces as geographic units, with color indicating estimated temperatures categorized into quintiles of the distribution. Higher color intensity corresponds to higher temperatures.

Data were described using means (standard deviation − SD) or proportions (%) and compared with the t-test or chi-squared test, as appropriate, and displayed in Table 1. Moreover, temperature data were graphically represented on a map (Figure 1). These and all subsequent analyses were conducted using Stata software, version 18 (https://www.stata.com).

**Table 1:** Characteristics of the study population by overweight status (n = 16,410).

Characteristics	Overweight				Total (n = 16,410)		p-value
	No (n = 5,268)		Yes (n = 11,142)				
	Mean	SD	Mean	SD	Mean	SD	
Age (years)	40.1	18.2	49.0	17.1	46.1	17.9	< 0.001
Variables	Count	%	Count	%	Count	%	
Sex							
Male	1,988	38	4,972	45	6,960	42	< 0.001
Female	3,280	62	6,170	55	9,450	58	
Education							
Incomplete primary school	365	7	1,282	12	1,647	10	< 0.001
Incomplete secondary school	1,656	31	4,250	38	5,906	36	
Complete secondary school	2,165	41	3,847	35	6,012	37	
Tertiary or university	1,082	21	1,763	16	2,845	17	
Physical activity							
Low	2,125	40	5,549	50	7,674	47	< 0.001
Moderate	1,985	38	3,855	35	5,840	36	
High	1,158	22	1,738	16	2,896	18	
Fruit/Vegetable consumption (≥ 5 servings/day)							
No	4,812	94	10,168	94	14,980	94	0.345
Yes	299	6	676	6	975	6	

SD: standard deviation.

#### Parallel multiple mediation models

The generalized structural equation model (GSEM) was performed in sequential steps (Table 2). The units of analysis comprised 15,955 observations after excluding 455 cases with missing data on fruit and vegetable consumption. First, a variance component model examining the relationship between ambient temperature (exposure as a continuous variable) and overweight (outcome, no/yes), with a latent contextual variable at the provincial/jurisdictional level, was fitted. Subsequently, a second model including sex (male/female), age (continuous), and education (incomplete primary school, incomplete secondary school, completed secondary school, and university/tertiary degree) as covariates (Model 2, Table 2) and a third model incorporating physical activity level and fruit/vegetable consumption (Model 3, Table 2) were fitted. Goodness of fit was assessed using the Akaike information criterion (AIC) and Bayesian information criterion (BIC). A multiple mediator model (Model 4, Table 2) was then adjusted considering physical activity level and fruit/vegetable consumption as mediators. Survey weights were not applied due to technical limitations in fitting multilevel models with GSEM in Stata, prioritizing the hierarchical specification required for the analysis.

**Table 2:** Sequential models examining the relationship between ambient temperature (continuous variable) and overweight (yes/no).

Overweight (outcome)	Crude model		Model 1		Model 2		Model 3		Model 4	
	Coefficient	95%CI	Coefficient	95%CI	Coefficient	95%CI	Coefficient	95%CI	Coefficient	95%CI
Mean 2m-air temperature (continuous)	-0.013	-0.027; 0.001	-0.013	-0.027; 0.001	-0.016	-0.032; -0.0003	-0.019	-0.034; -0.004	-0.019	-0.034; -0.004
Age (continuous)	0.030	0.028; 0.032			0.031	0.028; 0.032	0.029	0.027; 0.031	0.029	0.027; 0.031
Sex (male as reference)										
Female	-0.290	-0.358; -0.223			-0.330	-0.399; -0.260	-0.365	-0.437; -0.293	-0.365	-0.437; -0.293
Education (university as reference)										
Incomplete primary school	0.743	0.602; 0.883			0.299	0.152; 0.446	0.269	0.119; 0.420	0.269	0.119; 0.420
Incomplete secondary school	0.423	0.327; 0.519			0.338	0.239; 0.438	0.317	0.216; 0.418	0.317	0.216; 0.418
Complete secondary school	0.059	-0.033; 0.152			0.222	0.126; 0.319	0.208	0.110; 0.306	0.208	0.110; 0.306
Physical activity (low level as reference)										
Moderate	-0.290	-0.365; -0.152					-0.168	-0.247; -0.090	-0.168	-0.247; -0.090
High	-0.567	-0.658; -0.476					-0.362	-0.460; -0.264	-0.362	-0.460; -0.264
Fruit and vegetable consumption (< 5 servings/day as reference)										
≥ 5 servings/day	0.049	-0.092; 0.191					0.025	-0.123; 0.173	0.025	-0.123; 0.173
Moderate physical activity (outcome)										
Mean 2m-air temperature (continuous)									-0.015	-0.023; -0.007
High physical activity (outcome)										
Mean 2m-air temperature (continuous)									-0.059	-0.068; -0.049
Fruits and vegetables consumption (outcome)										
Mean 2m-air temperature (continuous)									0.020	0.005; 0.035
Area-level clustering			Variance	SE	Variance	SE	Variance	SE	Variance	SE
Province			0.023	0.008	0.030	0.011	0.026	0.010	0.026	0.010

95%CI: 95% confidence interval; AIC: Akaike information criterion; BIC: Bayesian information criterion; SE: standard error.

Note: Model 1: AIC = 20546, BIC = 20569.12; Model 2: AIC = 19464.92, BIC = 19526.56; Model 3: AIC = 18858.38, BIC = 18942.83; Model 4: parallel multiple mediation models.

**Figure 2:**
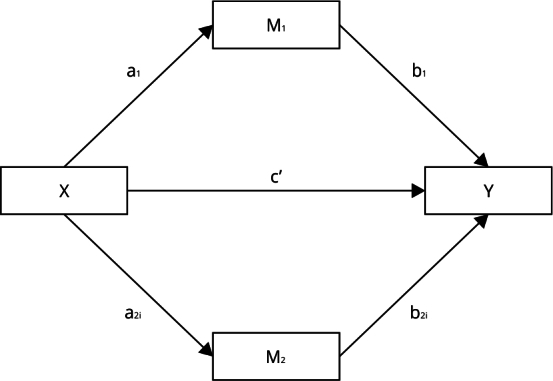
Parallel multiple mediator model of the relationship between ambient temperature and overweight.

Note: the figure depicts the direct effect of mean 2m-air-temperature (X) on overweight (Y), with c coefficient; and the mediating variables (M_1_ and M_2_) fruit/vegetable consumption (≥ 5 servings/day, no/yes), and physical activity level (i), respectively, with a and b coefficients.

The selected model (Figure 2) considers a direct effect of mean 2m-air temperature (X, continuous) on overweight (X→Y; c coefficient), with Y assumed to follow binomial distribution. Fruit/vegetable consumption and PA level (low/moderate/high) were identified as mediators (M stage: M_1_ and M_2_, respectively) in this relationship (Outcome stage: X→M_1_→Y; a_1_.b_1_ for the indirect effect of M_1_; and X→M_2_→Y; a_2_.b_2_ for the indirect effect of M_2_) (Figure 2). 

Moreover, models were fitted using the median 2m-air instead of the mean, without changes in results.

#### Metrics for comparing indirect effects

To compare the magnitude and effects of mediator variables, the metrics proposed by Coutts & Hayes ^25^ were applied, particularly the raw difference, estimated as the difference between the indirect effects to be compared, in this case: *a*_*1*_ *b*_*1*_ *− a*_*2i*_ *b*_*2i*_ .

Since physical activity level (M_2_) was categorized into three levels, two mediation pathways (i) were created corresponding to moderate (M_21_) and high (M_22_) physical activity levels, with low physical activity as the reference category. These pathways were then compared to the fruit/vegetable consumption (≥ 5 servings/day) mediation mechanism.

A bootstrap confidence interval was applied to the sampling distribution of the estimated direct and indirect effects, as well as to the difference between indirect effects ^26^ and total effects. The 95% confidence interval (95%CI) was built by bootstrapping the raw difference with a 10,000-unit random sample and 5,000 replications. The histogram of the bootstrap distribution was graphically displayed. Evidence of a difference in magnitude between indirect effects was found if the confidence interval did not include zero ^25^.

To further probe the effects of direct and indirect pathways on the outcome, obesity (BMI ≥ 30) was analyzed using the same methodological steps (Table 3).

**Table 3:** Coefficients and effects estimated, and comparisons of indirect effects using bootstrapping techniques for obesity as outcome.

Mediation paths	Coefficients	Value	SE	95%CI
X → M_1_	a_1_	0.020	0.007	0.005; 0.035
M1 → Y	b_1_	0.018	0.072	-0.123; 0.160
X → M_21_	a_21_	-0.015	0.003	-0.023; -0.007
M_21_ → Y	b_21_	-0.234	0.038	-0.310; -0.159
X → M_22_	a_22_	-0.059	0.004	-0.068; -0.049
M_22_ → Y	b_22_	-0.413	0.051	-0.514; -0.311
Direct effects				
X → Y	c	-0.021	0.005	-0.032; -0.009
Indirect effects				
X → M_1_ → Y	a_1_ b_1_	0.0003	0.002	-0.003; 0.004
X → M_21_ → Y	a_21_ b_21_	0.003	0.001	0.0007; 0.006
X → M_22_ → Y	a_22_ b_22_	0.024	0.004	0.015; 0.033
	Estimators	Value	Boot SE	95% Boot CI
Total effects				
Direct plus Path _1_	c + a_1_ b_1_	-0.020	0.005	-0.031; -0.009
Direct plus Path _21_	c + a_21_ b_21_	-0.017	0.005	-0.028; -0.006
Direct plus Path _22_	c + a_22_ b_22_	0.003	0.006	-0.009; 0.016
Metrics of difference				
Raw difference M_1_ vs. M_21_	a_1_b_1_ − a_21_b_21_	-0.030	0.002	-0.001; 0.008
Raw difference M_1_ vs. M_22_	a_1_b_1_ − a_22_b_22_	0.024	0.005	0.014; 0.034

95%CI: 95% confidence interval; 95% Boot CI: 95% bootstrap confidence interval; Boot SE: bootstrap estimate of standard error; M_1_: fruit/vegetable consumption of ≥ 5 serving/day; M_21_: moderate level of physical activity; M_22_: high level of physical activity; X: 2m-air temperature; Y: obesity.

## Results

Table 1 shows participant characteristics (n = 16,410). The mean age (SD) was 46.1 (16.9) years. Women represented a higher proportion in both groups, without (62%) and with overweight (55%). Statistical associations were observed between overweight status and both sex and educational level (p < 0.001), with a higher percentage of individuals with low educational attainment (incomplete secondary school or less) among those with overweight compared to those without (50% vs. 38%, respectively) (Table 1). This is consistent with the adjusted models, in which higher education was associated with lower odds of overweight, while female sex showed an inverse association (Table 2).

A statistically significant association was also observed with physical activity: 60% of participants without overweight had moderate or high physical activity levels, whereas about half of those with overweight had low physical activity levels (Table 1). Most participants who consumed fewer than five servings/day of fruits/vegetables were overweight (94%), although these percentages were not statistically different. Figure 1 shows a map illustrating the unequal distribution of mean ambient temperature across Argentina.

**Figure 3:**
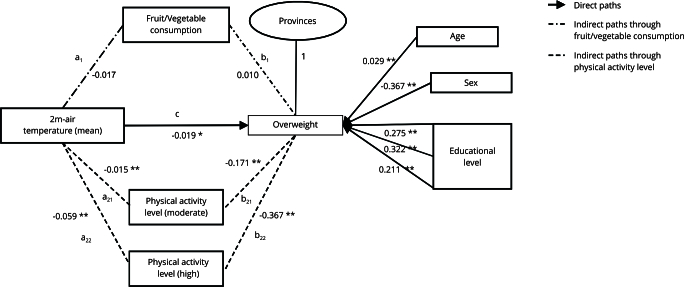
Results of the multiple mediator model of the temperature-overweight relationship in Argentina, comparing mediation pathways of fruit/vegetable consumption and physical activity levels, using a generalized structural equation model (GSEM).

Results from the parallel multiple mediator model, adjusted for age, sex, and education, are summarized in Table 2 and graphically displayed in Figure 3. A direct statistically significant negative effect of 2m-air temperature on overweight was observed (-0.019, 95%CI: -0.034; -0.004). A unit increase in 2m-air temperature increased the log odds of fruit/vegetable consumption (a_1_ = 0.020, 95%CI: 0.005; 0.035) while decreasing the log odds of moderate (a_21_ = -0.015, 95%CI: -0.023; -0.007) and high (a_22_ = -0.059, 95%CI: -0.068; -0.049) physical activity levels, compared to low fruit/vegetable consumption and low physical activity, respectively. Moreover, consuming ≥ 5 servings/day of fruits/vegetables showed a non-significant increase of the log odds of overweight (b_1_ = 0.025, 95%CI: -0.123; 0.173), while moderate (b_21_ = -0.168, 95%CI: -0.247; -0.090) and high (b_22_ = -0.362, 95%CI: -0.460; -0.264) physical activity categories were inversely and significantly associated. 

The specific indirect effect of a unit increase in 2m-air temperature for fruit/vegetable consumption had only a minimal effect on overweight (a_1_b_1_ = 0.0005; 95%CI: -0.003; 0.004) (Table 4). The specific indirect effects of 2m-air temperature for physical activity were a_2_b_21_ = 0.002 (95%CI: 0.0002; 0.0004) for moderate physical activity and a_2_b_22_ = 0.021 (95%CI: 0.012; 0.030) for high physical activity, both of which increased overweight, particularly high physical activity (Table 4). Additionally, in provinces with higher average annual temperatures, these effects were more pronounced (data not shown).

**Table 4:** Coefficients and effects estimated, and comparisons of indirect effects using bootstrapping techniques for overweight as outcome.

Mediation paths	Coefficients	Value	SE	95%CI
X → M_1_	a_1_	0.020	0.007	0.005; 0.035
M_1_ → Y	b_1_	0.025	0.075	-0.123; 0.173
X → M_21_	a_21_	-0.015	0.003	-0.023; -0.007
M_21_ → Y	b_21_	-0.168	0.040	-0.247; -0.090
X → M_22_	a_22_	-0.059	0.004	-0.068; -0.049
M_22_ → Y	b_22_	-0.362	0.049	-0.460; -0.264
Direct effects				
X → Y	c	-0.019	0.007	-0.034;-0.004
		Value	Boot SE	95% Boot CI
Indirect effects				
X → M_1_ → Y	a_1_ b_1_	0.0005	0.002	-0.003; 0.004
X → M_21_ → Y	a_21_ b_21_	0.002	0.001	0.0002; 0.0004
X → M_22_ → Y	a_22_ b_22_	0.021	0.004	0.012; 0.030
	Estimators	Value	Boot SE	95% Boot CI
Total effects				
Direct plus Path _1_	c + a_1_ b_1_	-0.019	0.005	-0.030; -0.007
Direct plus Path _21_	c + a_21_ b_21_	-0.016	0.005	-0.027; -0.005
Direct plus Path _22_	c + a_22_ b_22_	0.002	0.006	-0.010; 0.015
Metrics of difference				
Raw difference M_1_ vs. M_21_	a_1_b_1_ − a_21_b_21_	0.002	0.002	-0.002; 0.006
Raw difference M_1_ vs. M_22_	a_1_b_1_ − a_22_b_22_	0.020	0.004	0.001; 0.030

95% Boot CI: 95% bootstrap confidence interval; Boot SE: bootstrap estimate of standard error; M_1_: fruit/vegetable consumption of ≥ 5 serving/day; M_21_: moderate level of physical activity; M_22_: high level of physical activity; X: 2m-air temperature; Y: overweight.

**Figure 4:**
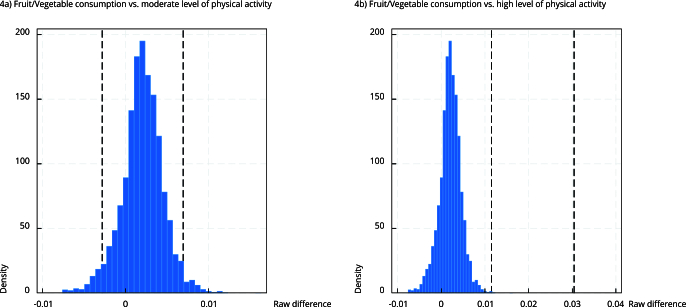
Bootstrap distributions of the raw differences between indirect effects of fruit/vegetable consumption vs. moderate level of physical activity and fruit/vegetable consumption vs. high level of physical activity.

Note: dashed lines indicate 95% bootstrap confidence interval endpoints.

Subsequently, the raw difference metric was applied to both indirect effects to test the equality of magnitude and value of the effects. The results of contrasting indirect pathways including fruit/vegetable consumption and moderate physical activity level were 0.002 (95%CI: -0.002; 0.006), whereas indirect pathways including fruit/vegetable consumption and high physical activity level were 0.020 (95%CI: 0.001; 0.030) (Table 4). Figure 4 shows the confidence intervals of the bootstrap distributions of the difference between fruit/vegetable consumption and moderate (Figure 4a) or high (Figure 4b) physical activity. As shown, the confidence interval of B (fruit/vegetable consumption vs. high physical activity) does not include zero.

## Discussion

The increasing prevalence of overweight and obesity worldwide requires a thorough examination of their multi-causal etiology ^27^ ^,^ ^28^. While the relationship between nutrition, physical activity, and overweight has been extensively studied ^29^, research exploring the relationship between climate variables and body weight remains limited, albeit with promising findings ^3^. Increased obesity rates and accelerated climate change represent global health challenges driven by lifestyle changes and human environmental modifications ^30^, with significant economic implications ^4^. This study estimated the effect of ambient temperature on overweight in the Argentine population aged 18 and older of 2018, while exploring the role of physical activity and fruit/vegetable consumption as mediating pathways in a parallel mediator model. Our findings showed that the effect of 2m-air temperature on overweight operates simultaneously through direct and indirect effects. However, the indirect pathway involving high physical activity levels was stronger and differed in magnitude from the fruit/vegetable consumption pathway.

As previously reported ^31^ ^,^ ^32^, our sample also revealed 67.9% of overweight participants, with higher prevalence in men than in women. Moreover, 38.14% of overweight people had not completed secondary education, similar to previous studies ^31^ ^,^ ^33^, and greater impact of extreme temperatures on more vulnerable socio-educational groups was also noteworthy ^34^. Moreover, from a syndemic perspective the negative effects of climate change are exacerbated by the interaction between chronic diseases and socioeconomic factors ^4^ ^,^ ^35^.

The impact of ambient temperature on energy expenditure and dietary intake has been explored, but its relationship with overweight remains unclear ^36^ ^,^ ^37^. We identified an inverse direct effect of 2m-air temperature on overweight, which contrasts with previous research. For instance, one study − which employed a design that did not consider mediating factors − suggested that elevate ambient temperature was linked to an approximately 5% increased likelihood of obesity ^2^. Another study reported a similar impact, particularly among girls and women ^3^. It has been proposed that higher atmospheric ambient temperature reduces adaptive thermogenesis and leads to less physical activity ^38^. Conversely, growing evidence indicates that humans expend energy on thermoregulation ^39^, and recent studies have shown that adults have functional brown adipose tissue depots ^40^ ^,^ ^41^ ^,^ ^42^ ^,^ ^43^ that respond to environmental temperature and exhibit seasonal variation. This result suggests a negative energy balance may occur at high ambient temperature, as appetite and intake decrease while energy expenditure increases ^42^ ^,^ ^44^ ^,^ ^45^. Differences in the climatic conditions of the studied populations could explain these contrasting results. We found an inverse relationship between temperature and moderate and high physical activity levels (versus low physical activity), though some studies have reported opposite results ^46^. A systematic review on climate change and physical activity indicated that this relationship is complex and multifaceted ^47^, showing that rising temperatures were associated with a net increase in active travel and leisure-time physical activity worldwide. However, this trend may reverse once a certain temperature threshold is exceeded. This latter point aligns with our results, considering our cross-sectional design and the use of geographically aggregated environmental temperature data. Therefore, the temperature trend in our study can be interpreted not as a continuous increase, but as a transition from cooler to warmer geographic areas. Argentina has significant climatic variations across regions, with annual mean temperatures ranging from 4.6-14.8°C in the south to 17.4-23°C in the north.

Some studies have examined the ambient temperatures food intake relationship using purchase records. While no specific association was found with fruit/vegetable consumption, results indicated a general negative effect of temperature on food intake ^7^. Although the connection between food choices and climate is not yet fully understood ^48^, it is well established that food choices are constrained by local food availability. The complexity of food selection process is related to its availability and accessibility ^49^. The ongoing global climate change and diet-related health crises are linked to food systems, environments, and consumer choices. Understanding these choices and the effects of climate change is essential for transforming the food system to benefit human and planetary health. In Argentina, contextual variables can determine the accessibility and availability of fruits/vegetables within food systems ^50^. Moreover, fruit/vegetable consumption is generally low, with few people reporting adequate consumption ^20^ ^,^ ^51^. This could explain the lack of association found between fruit/vegetable consumption and overweight. Incorporating additional dietary components in future analyses would offer a more comprehensive understanding of the intersection between climate, diet, and overweight.

The mediator pathways of fruit/vegetable consumption and physical activity level in the temperature-overweight relationship showed a significant positive effect for the physical activity paths. It is difficult to compare our results with others since the literature using this technique in health sciences is limited. Other approaches have been applied instead, such as analyses of the interaction between physical activity and diet, which suggest that a combination of increased physical activity and a healthy diet reduces obesity risk ^52^. Nevertheless, it remains unclear which of these factors has the strongest effect on the risk of obesity ^53^ ^,^ ^54^, as the association between obesity and sedentary behavior, low phyzical activity, alcohol consumption, and smoking habits is still inconclusive. 

To characterize the two indirect pathways, we used the raw difference to assess the magnitude of their effects. High physical activity was stronger in magnitude than the fruit/vegetable consumption. The application of mediation analyses, combined with metrics to compare indirect effects, is a valuable methodological strategy for investigating the underlying mechanisms in the complex ambient temperature-health relationship. This approach also provides important insights for public policy interventions ^55^.

It is worth noting that the total effect of both ambient temperature and physical activity, as well as fruit/vegetable consumption, is closer to zero than the direct effect, with mediator paths of opposite signs. This constitutes an inconsistent mediation, indicating that the indirect pathways counteract the direct effect ^56^. 

Some methodological issues must be considered. Our study addresses the climate-health relationship, emphasizing the limited development of science-based climate policies in South American countries and a lack of studies on nutrition ^17^. The ENFR employs a complex random sampling scheme and a hierarchical structure integrating environmental and individual data. However, this survey only collected fruit/vegetable consumption data focused on urban populations, making it challenging to thoroughly analyze diet-mediated pathways or understand how temperature-overweight associations might differ in rural areas. Moreover, the low prevalence of adequate fruit and vegetable consumption in the sample further constrained the detection of meaningful associations. A prospective design would strengthen this cross-sectional study to assess the causal relationship between exposure and outcome, considering mediation variables. The effect of ambient temperature may have been underestimated in certain regions, as satellite-derived air temperature data was aggregated at a broad geographic scale (province) and averaged annually to align with individual-level data. However, including a contextual latent variable in our final model allowed us to account for unobserved heterogeneity at the provincial scale. 

Misclassification bias of the exposure, particularly for fruit/vegetable intake, could be present. However, if present, this misclassification would likely be non-differential and bias toward the null hypothesis. Ambient temperature, physical activity, and BMI were assessed in a reliable way. Although the reliability of measured ambient temperature, it was averaged by month and year. We conducted several analyses by grouping provinces by geographical and climatic zones but did not find significant estimated differences.

Moreover, there may be uncontrolled confounding factors, particularly large spatial variations in poverty levels, a known key risk factor for obesity ^57^. To minimize this, we included “education” as a proxy for socioeconomic status. Nevertheless, residual confounding may persist, especially from unobserved factors such as characteristics of the built environment or disparities in access to healthy food and opportunities for physical activity.

## Conclusion

Our study showed that ambient temperature impacts overweight by two indirect effects that contribute to a decrease in overweight. The indirect high physical activity pathway was stronger and different in magnitude from the fruit/vegetable consumption pathway. Although further research is required to consolidate these findings, our results indicate that the identified risk factors significantly impact the development of overweight. Given that obesity is a major public health issue due to its association with NCDs and all-cause mortality, public policies aimed at preventing overweight ought to consider climate change and prioritize the most vulnerable populations ^58^. As highlighted, there is a need to improve food availability and access, especially in the most vulnerable regions ^49^, while recognizing the health and environmental co-benefits of promoting sustainable diets and food systems. The evidence obtained also suggests placing greater emphasis on physical activity patterns, focusing interventions on promoting physical activity in warm climates by increasing opportunities and improving environmental quality through safety, amenities, and facilities ^59^.

## Data Availability

The research data are available upon request to the corresponding author.
